# Factors in the Use of Workplace Health Promotion on Back Health. Results of the Survey “German Health Update”

**DOI:** 10.3389/fpubh.2021.638242

**Published:** 2021-04-12

**Authors:** Sophie Hermann, Anne Starker, Raimund Geene, Susanne Jordan

**Affiliations:** ^1^Institute of Medical Sociology and Rehabilitation Science, Charité – Universitätsmedizin Berlin, Berlin, Germany; ^2^Unit Health Behaviour, Department of Epidemiology and Health Monitoring, Robert Koch Institute, Berlin, Germany; ^3^Alice Salomon Hochschule, University of Applied Sciences and Charité – Universitätsmedizin Berlin, Berlin School of Public Health, Berlin, Germany

**Keywords:** workplace health promotion, employees, prevention, self-reported low back health, physical activity, socio-demographic factors, health and work-related factors, German health update (GEDA 2014/2015-EHIS)

## Abstract

**Background:** The influence of the working environment on the back health of employees is well-documented. Many companies have begun to offer employees access to services to promote back health. Factors affecting the use of these offers at the population level have received little investigation to date. The current study examined the socio-demographic factors, physical activity and health-related factors, and work-related factors associated with the use of offers of workplace health promotion for back health in Germany.

**Materials and Methods:** In the representative population-based cross-sectional survey “German Health Update” (GEDA 2014/2015-EHIS) conducted by the Robert Koch Institute, 12,072 employees aged 18–64 years old were surveyed from November 2014 to July 2015 regarding the use of back health services in their companies. In addition to socio-demographic factors, the survey examined working hours, physical activity in leisure time, health awareness, and subjective complaints in the lower back or other chronic back problems in the last 12 months. The interaction of these factors with the utilization of back health services was tested using multiple logistic regression models.

**Results:** Women used back health services more often than men (women: 25.5%; men: 18.1%). Female gender was associated with part-time employment (OR 0.72) and a strong to very strong level of health awareness (OR 1.40). Male gender was associated with age between 30 and 44 years (OR 1.99) and 45–64 years (OR 2.02), low socioeconomic status (OR 0.48), endurance activity of <2.5 h per week (OR 0.62), and absence of lower back pain or other chronic back conditions for the last 12 months (OR 0.48).

**Conclusion:** The present study is the first to provide findings regarding the factors associated with the utilization of workplace health promotion to promote back health at the population level, and from the perspective of employees in Germany. The results revealed that the relevant factors for participating in offers differ for women and men. To reach more employees, workplace health promotion offers for back health should be designed specifically for each individual, considering gender and age, working hours, health awareness and behavior, and health state.

## Introduction

A previous systematic review highlighted the influence of the working environment on the back health of employees (1). In context of the workplace, scientific studies indicate especially biomechanical overload due to patients' manual handling as related risk factors for chronic back pain. These include, for example, lifting heavy loads, working in a stooped or twisted posture, vibration, one-sided postures, and repetitive, unilateral movements (2). Also, the importance of psychological risk factors for the development and chronification of musculoskeletal disorders is being recognized and supported by empirical findings. In fact, back complaints occur to a significant extent in industries where light or no physical work is predominant. This indicates the presence of work-related psychosocial conditions such as low job satisfaction, monotonous work, and social conflicts at work as risk factors (3). Furthermore, fear of movement (also known as kinesiophobia) and catastrophizing may influence the development of chronic discomfort and lower performance in the work environment, although these issues are poorly studied (2, 4, 5). Thus, physical activity, ergonomics in the workplace, stress, job satisfaction, social relationships, and company conditions have been identified as important factors in the prevention of back diseases (Medical Service of the German National Association of Health Insurance Funds (MDS) and the National Association of Statutory Health Insurance Funds (6).

Back pain is the second most common individual diagnosis, accounting for 5.9% of cases of incapacity to work and 6.0% of days of incapacity to work (7). At the same time, many companies and health insurance funds have recognized the potential of changes in the working environment to contribute to maintaining the health of employees. Beyond the legally required occupational safety measures, companies are increasingly offering additional measures to promote back health for their employees (6). These and all other joint measures taken by employers, employees, and society to improve health and well-being at work are referred to as Workplace Health Promotion (WHP) (8). Regarding concrete interventions, workplace health promotion can focus on the improvement of working conditions (e.g., by redesigning the work environment as well as the processes and communication structures in the company to improve the health of employees). In addition to the structures in the company, individual measures can also be directed at the health behavior of the employees. Previous studies suggest that combined interventions addressing behavior and conditions are superior to isolated behavioral or even relational preventive measures in terms of their effectiveness and efficiency (9).

The current study focused on behavioral WHP measures for back health such as back school and back exercises. In German companies, these are the most frequently offered individual measures (6). In addition, surveys of employees in Germany show that in the evaluation of all measures for WHP, back training is the most important offer for 65.7% of respondents (10). The overriding objectives of behavioral preventive back health measures are typically to promote exercise for employees in the workplace and to strengthen employees' individual skills and resources in dealing with stress (11). The design and evaluation of the measures involve various challenges, including the often-unclear causes of back pain, the multidimensionality of the identified risk factors and the high variability of frequently recurring symptoms. Thus, the respective approaches applied in back school courses or back exercises are different, ranging from device-supported training to endurance-oriented programs such as running and (Nordic) walking or compensatory gymnastics and breaks from exercise in the workplace to behavioral training and learning relaxation techniques (3). The evidence for different approaches varies with mostly small to moderate effects on their health-promoting effects. For example, this applies to mobilization and stretching programs (12). Metastudies have reported that programs designed to increase physical activity generally reduce both absenteeism due to musculoskeletal disorders and the incidence (number of new cases) and prevalence (incidence of disease) of back disorders (7). However, the existing research is inadequate, and comparable study results are not sufficient to draw firm conclusions about the effectiveness of the various behavioral preventive WHP approaches for promoting back health among employees. Thus, there is still an urgent need for further research (11).

According to the German Federal Statistical Office, in Germany in 2018, 72.1% of women and 79.6% of men of working age were employed (13). In the working environment, it is possible to reach target groups who rarely make use of individual prevention services (e.g., men, young and socially disadvantaged people) (14, 15). Although the number of WHP offers for back health has exhibited a slight upward trend in recent years, data from various surveys of employees show that the participation of employees in company sports and exercise programs, as well as back health programs, remains at a relatively low level (6, 10).

The success of WHP measures for promoting back health, in the sense of lasting change in health behavior at the population level, as well as group-oriented design of offers, depends on individual factors (e.g., age, gender, or health awareness) and contextual characteristics (e.g., working hours) of participants and non-participants. The utilization of WHP measures is typically assessed using data provided by surveys with employers or the annual prevention report of the National Association of Statutory Health Insurance Funds in Germany, which lists the WHP services supported by the insurance funds (6, 16, 17). In addition to inhouse offers and services provided by private providers, these account for the majority of the total WHP offers provided in Germany. Less frequently, data on the prevalence of WHP measures have been obtained from company employee surveys or population-based surveys of the economically active population (10, 18). The actual use and perception of WHP offers from the perspective of employees thus represents a previously neglected field of research (19). Available results are rarely detailed and provide little insight into the nature of the offerings or the concrete composition of the respective participant groups. In view of the high percentage of the employees with back pain; however, it seems necessary to fill existing gaps in knowledge about factors that influence the utilization of WHP services for back health.

Our study aimed to fill the gap in knowledge about factors that influence the use of workplace health promotion (WHP) offers for back health on the population level, so that future WHP offers can be tailored to the needs of the population. We use a population wide survey on workplace health promotion with data from the perspective of employees, which have been rarely used so far and should allow population wide conclusions. To better understand the factors that are associated with WHP utilization on the population level, we considered factors being of relevance in previous studies on health promotion and prevention, especially on the promotion on back health. At first, these are sociodemographic and -economic factors as age, gender as well as socioeconomic status which have shown to be associated with the use of different individual prevention offers (20–24). Employment status and working hours can be limiting or promoting factors for the participation in WHP (25–27) so we included these employment factors in our analyses. Health behavior in form of “physical activity,”, and “health awareness” have been proven to be positive associated with the use of prevention programs (2, 14, 20, 21). At last, back health by the factor “subjective complaints in the lower back or other chronic back conditions” was considered in the analyses, due to results that health conditions are associated with the use of health promoting and prevention measures (17, 28–30). By analyzing these factors in an overall model, we aimed to identify the factors that should be considered in the development of WHP-offers promoting back health in the population in a targeted manner.

## Materials and Methods

### Database

Data from the survey “German Health Update” (GEDA 2014/2015-EHIS) were used to analyze the association between the utilization of company offers for back health and selected determinants of back health (Table 1). This survey used web-based and paper-and-pencil questionnaires and was carried out between November 2014 and July 2015 as part of health monitoring by the Robert Koch Institute. The sample is based on statistics from the residents' registration office using a two-stage cluster procedure. The population of the study comprised persons aged 18 years and older with permanent residence in Germany. The topics of the survey are divided into constant core modules on health-relevant issues and a flexible topic in which current public health-relevant focal points are included. In total, 24,016 questionnaires were completed in the study: 10,723 web-based (44.6%) and 13,293 paper-pencil questionnaires (55.4%). The response rate (i.e., the ratio of completed interviews to the total number of people contacted from the population; “response rate 3,” American Association for Public Opinion Research) was 26.9% (women 27.5%, men 25.3%). A detailed description of the study methodology was published elsewhere (31, 32).

**Table 1:** Description of the analysis sample (employees aged between 18 and 64 years); Database: GEDA 2014/2015-EHIS.

		***n* (unweighted)**	**% (weighted)**	**95% CI**
Sex	Women	6.610	47.5	46.4–48.6
	Men	5.462	52.5	51.4–53.6
Age	18–29 years	1.662	15.8	15.0–16.7
	30–44 years	4.122	34.1	33.0–35.1
	45–64 years	6.288	50.1	49.0–51.2
Socioeconomic status (SES)	Low	1.418	15.2	14.4–16.1
	Middle	6.700	60.9	59.9–62.0
	High	3.951	23.8	23.0–24.7
	Missing	3		
Employment status	White-collar worker	9.200	73.3	72.2–74.2
	Blue-collar worker	1.795	19.5	18.6–20.4
	Civil servant (also candidate)	1.077	7.3	6.8–7.8
	Missing	0		
Working hours	Part-time	3.681	28.2	27.3–29.2
	Full-time	8.391	71.8	70.8–72.7
	Missing	0		
Minimum 2.5 h of moderately strenuous endurance activities per week	Yes	5.601	45.6	44.5–46.7
	No	6.239	54.4	53.3–55.5
	Missing	232		
Minimum 2 days a week of muscle strengthening activities	Yes	3.420	28.2	27.2–29.2
	No	8.600	71.8	70.8–72.8
	Missing	52		
Health awareness	Very strong	813	6.6	6.1–7.2
	Strong	4.590	36.1	35.1–37.1
	Moderate	5.757	49.3	48.2–50.4
	Less strong	770	6.9	6.4–7.6
	Not at all	100	1.1	0.8–1.3
	Missing	42		
Health awareness (aggregated)	Strong—very strong	5.403	42.7	41.6–43.8
	Less strong—not at all	6.627	57.3	56.2–58.4
Subjective complaints in the lower back or other chronic back problems in the last 12 months	Yes	4.259	37.1	36.1–38.2
	No	7.406	62.9	61.8–63.9
	Missing	407		

*CI, confidence interval*.

### Outcome Variable

The present study used information provided by the respondents regarding the use of company offers for back health. We first recorded respondents' knowledge of back health offers using an initial question: “Did your company/enterprise offer back health services (e.g., back school, back gymnastics) in the last 12 months?” (answer categories: yes/no/don't know). If the respondent answered yes, the next question asked was: “Have you taken advantage of this offer?” (answer categories: yes/no). To improve the comparability of the data, items from similar studies in Germany were adopted or adapted to GEDA 2014/2015-EHIS accordingly. These studies were carried out in recent years by the scientific institute of 11 regional health insurance funds (WIdO) and the Initiative Health and Work (iga) using representative telephone surveys of employees, mostly in connection with questions about work stress, occupational safety, and health (10, 33).

### Predictor Variables

The selection of variables included in the analyses was based on the results of existing research on factors associated with utilization, particularly studies examining offers for workplace health pro-motion and prevention, as well as the promotion of back health in the workplace, with the main focus on research results for Germany (1, 33–36). A number of relevant factors were identified: demographic characteristics (gender and age), socioeconomic status (SES), physical/sporting activity in leisure time and for locomotion, health awareness and subjective complaints in the lower back or other chronic back problems in the last 12 months.

### Demographic Characteristics

The following analyses considered female and male participants aged between 18 and 64 years old at the time of the survey. Participants were divided into three age groups: 18–29, 30–44, and 45–64 years.

### Socioeconomic Characteristics

Social differences in health of the respondents were analyzed by their SES. SES was calculated based on information about school education and vocational qualifications, occupational status, and needs-weighted net household income. Based on a points-sum index, in which the three indicators were equally weighted, a distribution-based differentiation of three status groups was performed, with the low and high status groups each comprising 20% of the population and the middle status group comprising 60% of the population (26).

### Employment Status and Working Hours

Information regarding employment status refers to the participants' subjective assessment of their current situation, in which participants were able to choose one of 13 given answers to the question: “Which life situation applies to you predominantly at present?” Individuals were classified as employed and included in the study if they chose one of the following answers: “I am employed full-time (also vocational training or self-employment, without part-time work for older employees),” “I am employed part-time (also vocational training or self-employment, without part-time work for older employees),” “I am marginally employed (e.g., 450-Euro job, mini-job).” These participants were then asked: “What is your main professional position in your main occupation?,” choosing from 12 predefined answer categories. For better comparability with similar national studies, only participants who answered “white-collar worker,” “blue-collar worker” or “civil servant (also candidate)” were considered in the following analyses. We excluded all inactive individuals as well as those who stated that they had completed a “voluntary social/ecological/cultural year” or were “voluntary military or federal volunteers.” In addition, participants who stated that they were “farmers” as their main occupation, “self-employed (with or without employees),” “helping company employees” or “trainees (including interns, volunteers)” were not considered.

The scope of employment was divided into two categories based on the information on employment status provided by respondents, as described above: “part-time employed” (also includes part-time workers and persons in partial retirement) and “full-time employed.”

### Physical Activity in Leisure Time and for Locomotion

Physical activity indicators are based on the World Health Organization (WHO) exercise recommendations for adults, which distinguish between “endurance activities” and “muscle strengthening activities” (37). Respondents are asked four questions about the length of time per week of moderately aerobic physical activity, sport or fitness in leisure time, walking and cycling for locomotion and the number of days per week of physical activity specifically for muscle strengthening. Details regarding indicator formation have already been published elsewhere (38). The following table shows the proportions of participants who spend a minimum of 2.5 h of at least moderately strenuous endurance activities per week (the first part of the WHO exercise recommendation) or muscle strengthening activities for at least 2 days a week (second part of the WHO exercise recommendation). The proportion of participants who fulfilled both parts of the WHO recommendation (2.5 h of endurance plus muscle strengthening activities twice per week) is also shown in the table.

### Health Awareness

Health awareness was measured by asking “How much do you generally pay attention to your health?” (very strong/strong/moderate/less strong/not at all) (39). For the evaluations, the combined answers “very strong/strong” and “moderate/less strong/not at all” are shown.

### Subjective Complaints in the Lower Back

The “Diseases and complaints” part of the questionnaire was used to record the 12-month prevalence of lower back complaints or other chronic back problems. In addition, it was determined whether these complaints had ever been diagnosed by a doctor. Respondents were asked to answer either “Yes” or “No” to the questions: “Have you had lower back problems or other chronic back problems within the last 12 months?” and “Has this ever been diagnosed by a doctor?.”

### Statistical Analyses

Analyses were performed with the Stata SE 15.1 statistical package. To correct deviations of the sample from the population structure (as of 31.12.2014) with regard to gender, age, district type and education, a weighting factor was calculated, whereby the district type reflects the degree of urbanization and corresponds to the regional distribution in Germany. A significant difference was assumed if the *p*-value calculated under consideration of the weighting and survey design was smaller than 0.05. In the following analyses, the frequencies and 95% confidence intervals (CI) of the participant groups will be presented differentiated by gender. Subsequently, logistic regression models were used to analyze the association between the dependent characteristics and utilization, also differentiated by gender. Odds ratios (OR) were used to interpret the relationships. For example, an OR of 2.00 for full-time employed women (reference group of part-time employed women) would indicate that the likelihood of full-time employed women participating in WHP offers for back health was twice as high as that for part-time employed women. The variables were gradually added to the model to identify relevant factors for each group. Model 1 includes demographic and socioeconomic characteristics (age, SES) and the level of employment. Model 2 additionally includes WHO-recommended physical activity (endurance and muscle strengthening activity) and health awareness. In model 3, data on subjective lower back complaints or other chronic back problems in the last 12 months were also considered. Statistically significant results (*p* < 0.05) are reported in the Results section.

## Results

### Sample Characteristics

Table 1 shows the characteristics of the analysis sample differentiated by demographic and socioeconomic characteristics (gender, age, SES) as well as by employment status and working hours. Table 1 also shows the distribution of the variables used in the other analyses: endurance activity for at least 2.5 h per week, muscle strengthening activity at least twice per week, health awareness and subjective lower back complaints or other chronic back problems in the last 12 months. The unweighted absolute frequencies as well as the relative frequencies are shown.

### Bivariate Analyses

A higher proportion of women (25.5%) than men (18.1%) used their company's offers for back health (Table 2) (*p* = 0.001). There were no significant differences between age groups for either gender. Among women, significant differences were found in the group comparisons for two of the potential predictor variables. Regarding to the level of employment, women who were employed part-time were less likely to take advantage of a WHP offer for back health than women who were employed full-time (*p* = 0.040). Furthermore, in terms of health awareness, women who stated that they were strongly or very strongly health-conscious took advantage of such services more frequently than women with a lower level of health awareness (*p* = 0.002). Regarding SES, endurance and muscle strengthening activity, as well as subjective lower back complaints or other lower back complaints in the last 12 months, there were no significant group differences in women. Among male workers, significant group differences were found for four of the potential predictor variables. The results revealed that men with low SES used WHP offers for back health more often (*p* = 0.018) than men with medium or high SES. In addition, men who followed the WHO recommendations for endurance activity (*p* = 0.004) and muscle strengthening activity (*p* = 0.013) used offers more frequently than men who did not follow the recommendations. Men also differed in their use of the company's services depending on whether they reported subjective complaints in the lower back or other chronic back problems. Men with complaints in the last 12 months participated in offers significantly more often than men without complaints.

**Table 2:** Use of company offers to promote the subjective back health of employees aged 18–64 years (*n* = 3,069; women: 1,468, men: 1,601) in the last 12 months; Database: GEDA 2014/2015-EHIS.

**Offers to promote back health**		**Women**			**Men**		
		**%**	**95% CI**	***P*-value**	**%**	**95% CI**	***P*-value**
Total		25.5	22.8–28.4		18.1	16.0–20.5	
Age	18–29 years	24.4	18.4–31.6	0.533	13.6	8.5–21.0	0.329
	30–44 years	23.8	19.6–28.6		18.7	14.8–23.4	
	45–64 years	27.0	23.0–31.4		18.9	16.3–21.9	
Socioeconomic status (SES)	Low	25.5	14.7–40.3	0.687	27.9	18.8–39.3	0.018
	Middle	26.3	20.9–30.1		18.3	15.4–21.5	
	High	23.6	19.8–27.9		15.4	12.7–18.5	
Working hours	Part-time	22.3	18.3–26.8	0.040	17.5	10.4–27.9	0.886
	Full-time	28.0	24.6–31.6		18.2	15.9–20.7	
Minimum 2.5 h of moderately strenuous endurance activities per week	Yes	26.6	22.9–30.2	0.320	21.0	18.0–24.4	0.004
	No	23.8	20.0–28.0		14.5	11.8–17.8	
Minimum 2 days a week of muscle strengthening activities	Yes	28.4	23.7–33.7	0.126	22.1	18.2–26.5	0.013
	No	24.1	21.1–27.4		16.2	13.7–19.0	
Health awareness	Strong—very strong Less strong—not at all	29.6 21.3	25.7–33.8 18.0–25.1	0.002	19.9 17.6	16.6–23.7 13.9–20.0	0.183
Subjective complaints in the lower back or other chronic back problems in the last 12 months	Yes	28.2	23.9–32.9	0.160	24.9	20.6–29.8	0.000
	No	24.1	20.6–28.0		14.7	12.3–17.6	

*CI, confidence interval. Related to all individuals who knew about offers to promote back health in their company*.

### Regression Analysis

Stepwise multivariate logistic regression revealed significant associations between individual predictor variables and the utilization of WHP offers for back health. The results for women are shown in Table 3 for each model, and the results for men are shown in Table 4. Among female employees, women who worked part-time were less likely to take advantage of offers than women working full-time (Table 3). Depending on the regression model, the OR was approximately 0.72 (e.g., Model 3: OR 0.72, 95% CI 0.53–0.98). In addition, a strong to very strong level of health awareness had a significant effect, with an OR of 1.39 in model 2 (95% CI 1.03–1.88) and 1.40 in model 3 (95% CI 1.04–1.89) compared with women with a lower level of health awareness. Women showed no significant effects of age, SES, endurance and muscle strengthening activity, or subjective lower back complaints.

**Table 3:** Associations between factors and the use of company offers to promote the subjective back health of employees in the last 12 months **for women** (odds ratios and 95% confidence intervals); Database: GEDA 2014/2015-EHIS.

**Offers to promote back health**	**Model 1 Odds ratio (95% CI)**	***P*-value**	**Model 2 Odds ratio (95% CI)**	***P*-value**	**Model 3 Odds ratio (95% CI)**	***P*-value**
**Age**						
18–29 years 30–44 years 45–64 years	Ref. 1.10 (0.70–1.75) 1.23 (0.83–1.98)	0.674 0.271	Ref. 1.20 (0.76–1.90) 1.25 (0.80–1.94)	0.437 0.323	Ref. 1.15 (0.72–1.83) 1.26 (0.81–1.96)	0.562 0.313
**SES**						
Low Middle High	Ref. 1.02 (0.51–2.06) 0.86 (0.43–1.73)	0.954 0.663	Ref. 0.98 (0.45–2.16) 0.83 (0.38–1.81)	0.965 0.634	Ref. 1.03 (0.46–2.31) 0.84 (0.38–1.89)	0.943 0.682
**Working hours**						
Full-timePart-time	Ref. 0.71 (0.52–0.96)	0.025	Ref. 0.72 (0.53–0.98)	0.035	Ref. 0.72 (0.53–0.98)	0.039
**Minimum 2.5 h of moderately strenuous endurance activities per week**						
Yes No			Ref. 0.91 (0.64–1.28)	0.577	Ref. 0.93 (0.66–1.32)	0.699
**Minimum 2 days a week of muscle strengthening activities**						
Yes No			Ref. 0.87 (0.64–1.19)	0.376	Ref. 0.89 (0.65–1.23)	0.483
**Health awareness**						
Less strong—not at all Strong—very strong			Ref. 1.39 (1.03–1.88)	0.029	Ref. 1.40 (1.04–1.89)	0.028
**Subjective complaints in the lower back or other chronic back problems in the last 12 months**						
Yes No					Ref. 0.84 (0.62–1.15)	0.276

*CI, confidence interval; Ref., reference group*.

**Table 4:** Associations between factors and the use of company offers to promote the subjective back health of employees in the last 12 months **for men** (odds ratios and 95% confidence intervals); Database: GEDA 2014/2015-EHIS.

**Offers to promote back health**	**Model 1 Odds ratio (95% CI)**	***P*-value**	**Model 2 Odds ratio (95% CI)**	***P*-value**	**Model 3 Odds ratio (95% CI)**	***P*-value**
**Age**						
18–29 years 30–44 years 45–64 years	Ref. 1.50 (0.82–2.74) 1.52 (0.89–2.60)	0.187 0.121	Ref. 1.99 (1.07–3.71) 2.02 (1.15–3.54)	0.030 0.014	Ref. 1.93 (1.02–3.66) 1.71 (0.96–3.07)	0.044 0.071
**SES**						
Low Middle High	Ref. 0.59 (0.34–1.03) 0.62 (0.26–0.81)	0.062 0.007	Ref. 0.67 (0.38–1.17) 0.48 (0.26–0.86)	0.160 0.015	0.68 (0.37–1.23) 0.53 (0.28–1.00)	0.203 0.051
**Working hours**						
Full-time Part-time	Ref. 0.91 (0.47–1.75)	0.768	Ref. 0.82 (0.41–1.63)	0.571	Ref. 0.85 (0.41–1.73)	0.642
**Minimum 2.5 h of moderately strenuous endurance activities per week**						
YesNo			Ref. 0.64 (0.45–0.90)	0.011	Ref. 0.62 (0.44–0.87)	0.006
**Minimum 2 days a week of muscle strengthening activities**						
Yes No			Ref. 0.77 (0.55–1.07)	0.120	Ref. 0.75 (0.54–1.05)	0.090
**Health awareness**						
Less strong—not at all Strong—very strong			Ref. 1.11 (0.80–1.54)	0.532	Ref. 1.11 (0.80–1.55)	0.517
**Subjective complaints in the lower back or other chronic back problems in the last 12 months**						
Yes No					Ref. 0.48 (0.34–0.69)	0.000

*CI, confidence interval; Ref., reference group*.

For men, significant results were shown for both age and SES, depending on the model. The likelihood of taking advantage of an occupational offer for back health was approximately twice as high for men in the 30–44 and 45–64 years age groups (model 2: OR 1.99, CI 1.07–3.71 or OR 2.02, CI 1.15–3.54) compared with men aged between 18 and 29 years. In model 3 no significant effects were found for the 45–64 years age group. Compared with men with low SES, the likelihood of utilization by men with high SES was reduced by 0.62 (95% CI 0.26–0.81) in model 1 and reduced by 0.48 in model 2 (95% CI 0.26–0.86). In model 3, SES no longer showed a significant effect. Non-compliance with WHO recommendations for endurance activity was associated with lower likelihood of utilization (e.g., Model 3: OR 0.62, 95% CI 0.44–0.87) as was the absence of lower back pain or other chronic back conditions for the last 12 months (Model 3: OR 0.48, 95% CI 0.34–0.69). The factors working hours, muscle strengthening activity at least twice a week, and health awareness were not relevant for men.

## Discussion

### Key Results

The present study revealed new insights into the factors associated with the use of company offers for promoting back health from the perspective of the employees in Germany. Data from GEDA 2014/2015-EHIS revealed that, depending on gender, socio-demographic and occupational factors, health awareness, physical activity, and subjective back health were associated with utilization. Thus, women who were employed full-time and women with a strong to very strong level of health awareness exhibited an increased likelihood of using a service to promote back health in their company. For male employees, other factors were found to be relevant. Men who were older than 29 years, those with low SES, those who performed at least 2.5 h per week active in endurance activities in their free time and/or for exercise, and those who have had lower back problems or other chronic back problems in the last 12 months were more likely to take advantage of WHP promoting back health.

### Limitations

The current study involved several limitations that potentially limit the validity of our analyses. All variables we examined were based on self-assessment data, which could potentially lead to memory-related distortions and a bias toward socially desirable responses. In addition, it is also possible that there was not a uniform understanding of WHP in the surveys, and that terms were interpreted differently. For example, information regarding the existence of WHP may have been influenced by employees failing to assign some measures to WHP, and instead assigning them to occupational health and safety, causing a failure to report WHP measures for back health. More detailed information regarding the specific content, duration, structure, intensity, and quality of the behavioral prevention measure was not available, meaning that, for example, a single instance of participation was also included in the calculations. The wording of the question “Was there an offer for back health in your company in the last 12 months” involves ambiguity, which could not be completely counteracted by the addition of “(e.g., back school, back gymnastics).” For example, specific strength or aerobic training or programs such as Pilates and yoga can also be offered to promote back health, but were not explicitly mentioned in the questionnaire and may therefore not have been considered by the respondents.

When interpreting the results, the type of study design should also be considered. The current study had a cross-sectional design that did not allow for causal conclusions to be drawn. In addition, some of the results had large confidence intervals, due to relatively small case numbers for certain subgroups, adding further uncertainty. Also to be considered is the low response rate of 26.9% of GEDA 2014/2015-EHIS. Analyses of the response rate showed differences by age and gender which were therefore included in the weighting factors of the survey that we used for data analyses to adjust the sample distribution to the reference standard for Germany. However, we cannot exclude the possibility that selection bias occurred at the different stages of the sampling procedure (a detailed description of this and further response rate analyses can be found in the detailed methodological reports of the GEDA 2014/2015-EHIS in Saß et al. (32) and Lange et al. (31). Apart from that, the response rate is within the current low range for population-based health surveys in Germany using the same response rate calculation method. In many countries, the survey response rates have continuously decreased in the last decades (40, 41). However, compared to others, for this study design a relatively high degree of representativeness can be assumed.

### Interpretation

Regarding the distribution by gender, the results revealed an overall pattern for the use of prevention measures: women exhibited a higher rate of usage (25.5%) compared with men (18.1%). In accord with this finding, the Absenteeism Report 2008 and the iga Report 12 also reported higher utilization of services by women (10, 33). The BIBB/BAuA Employment Survey did not reveal significant gender differences for any of the measures, possibly because of the lack of differentiation between various thematic offerings (42). Data from the annual prevention reports of the National Association of Statutory Health Insurance Funds in Germany revealed that WHP promotion by health insurance funds is increasingly common in companies with a higher proportion of men, potentially enabling more men to be reached. However, the report also shows that, for most WHP measures, female employees ultimately have a higher utilization rate than male employees (6). This finding is supported by a review by Robroek et al. reporting that women had a higher likelihood of utilization than men (OR: 1.67 95% CI 1.23–2.27) (36). Beyond the workplace setting, studies in Germany have reported that statutory health insurance funds reach considerably more women than men with their behavioral prevention offerings for general prevention (6, 21, 22, 43, 44). One potential reason for the higher utilization by female employees is that women are generally more health-conscious and are more likely to show health-promoting behavior and/or lower risk behavior than men in many areas (14). For example, regarding the use of health services by women, a higher level of sensitivity to the body and health and a greater willingness to accept help have been reported (35). For men, often the manifestation of an illness or a perceived burden of suffering, such as pain, is required for the use of medical services to reach the same extent as that of women (20).

In investigating the causes of gender inequality, particularly in the context of the use of WHP, factors on the supply side should also be considered, rather than focusing exclusively on the demand side. For example, increased usage in female employees may arise from the gender-neutrality of offers, with fewer offers being specifically designed for men (45). The current statutory health insurance funds prevention report for the reporting year 2018 shows that only 5% of WHP offerings were targeted specifically to women, and 4% were targeted to men (6). Thus, the design, targeting and availability of current back health care offers may appeal more to people with the abovementioned attitudes, which are more common among women, and this may lead to better outcomes.

The current findings revealed that age was not associated with the use of the WHP offers for back health among female employees. Other studies have reported contradictory results regarding age in women, finding both higher and lower participation rates among older female employees (36). For male employees, however, age was a relevant factor in the current study. According to the results, men in the 30–44 and 45–64 years age groups were twice as likely to take advantage of a WHP offer for back health compared with those aged 18–29 years old. This could indicate that the offers are less tailored to the needs of younger men. As discussed in the previous section, it is often the manifestation of an illness or a perceived psychological strain that causes men to make use of medical services or preventive health care. Because chronic musculoskeletal disorders and stress experienced at work typically increase with age, this could provide a further explanation for the finding that younger men in particular were less likely to address health in the absence of more serious physical or psychological stress symptoms (22). Previous studies have not revealed the extent to which offers are also age- and gender-sensitive and which factors are associated with the use of WHP offers for back health among men of different ages.

As with age, SES was not associated with the use of back health services among female respondents. In contrast, male employees with low SES were more likely to make use of the services compared with those with high SES. In addition, this effect weakened when interacting with the factor of subjective lower back complaints or other chronic back problems in the last 12 months. This finding was in accordance with the gender and utilization results for men discussed above. However, interpreting these results is difficult because we are not aware of any comparative data from other studies of gender-specific differences in SES and the use of back health offers in workplace settings. Overall, however, the current results for both women and men should be viewed positively, as they did not confirm the findings of previous population-based studies for Germany. Several previous studies examining contextual factors have reported that women and men with high SES more frequently make use of health promotion and prevention programs and training opportunities (14, 44). These previous findings indicate that reducing socially induced health inequalities is an important challenge for public health (44). Low SES and low levels of education are considered to be indicators of social disadvantage and are associated with risk factors and poorer health. Although there is a need for prevention in all SES groups, the need is greater among people with low SES (24). The present study indicates that WHP offers for the promotion of back health have a valuable potential to reach employees across SES groups. This potential should be utilized to a greater extent in the future.

Regarding working hours, women who worked full-time were more likely to take advantage of offers to promote back health in their company compared with women working part-time. For men, employment level did not have a significant effect. The possible associations between the actual working hours of women and men in part-time employment and the extent to which they were utilized could not be deduced from the current study data. In general, other studies have indicated that employees in precarious employment situations (part-time/limited term/temporary) are less likely to take advantage of WHP interventions than employees in full-time, permanent, or non-temporary employment (35). When interpreting the results of female employees, it should be considered that there are pronounced differences in working hours between women and men in Germany (27). A significantly higher proportion of women work part-time, primarily in the family phase, potentially explaining why the factor of employment volume was only associated with utilization among female employees.

In the current study, physical activity was understood in terms of two indicators: “endurance activities” and “activities to strengthen muscles.” These indicators showed no relevant associations with utilization among female employees. Men who performed endurance activity for at least 2.5 h per week; however, were more likely to utilize WHP offers for back health. This result corresponds to central assumptions of known health behavioral patterns not specifically referring to WHP. These assumptions suggest that measures to promote physical activity are more frequently taken up by population groups that are already physically active (46). In addition, the results of other studies outside the WHP context have suggested that men are generally more often and more intensively physically active in their leisure time than women (37). One potential explanation for the association between physical activity and the use of WHP back health services, particularly among male employees, is that they may feel less attracted to the content and setting of back school or back exercises in the workplace. Thus, unlike women, for whom the indicators for physical activity were not associated with utilization, men who are not very physically active may not have felt that WHP offers for back health addressed their needs. Comparable analyses of other studies in the company context, particularly for male employees, are not available.

A previous study reported that the use of preventive measures is associated with health awareness (14). This association was also found in the present study, but only revealed significant effects among female employees. Although there are was no similar pattern for men, women with a strong to very strong level of health awareness were more likely to advantage of offers to promote back health compared with women with a low level of health awareness. Overall, health behavior can be explained primarily based on various subjective expectations, some of which have been empirically confirmed. For example, the expectation that individual actions can have positive effects on health influences health behavior and thus the use of preventive measures (45, 47). However, because previous studies of health awareness and health-related behavior have generally been conducted outside the working environment, it remains unclear whether and to what extent the described cause-and-effect relationships of health behavior are valid for predicting health-related actions in the workplace, particularly the use of WHP offers to promote back health.

Among men, subjective complaints about the lower back or other chronic back problems within the last 12 months were associated with the use of the services for the promotion of back health. This result was not observed for female employees. A previous study reported that, compared with healthy employees, groups who perceive their own health status as “poor” perform less sport (37), but more often take advantage of company prevention and health promotion programs (29). A perceived level of suffering or perceived “vulnerability” and the “risk” of limitations due to back pain or ailments are thus likely to motivate male employees in particular to take advantage of back schools or back exercises in the workplace. Such processes are also assumed to be an important factor in various models of behavioral change, such as the health-belief model (28) and the social-cognitive process model of health-related action (30).

### Generalisability

Our study design enables statements about WHP offers on back health for adult employees in Germany. We collected date on the use of measures at the population level with a representative sample, with the ability to include information about non-participants in the analyses. The factors examined – age, gender, social status, health awareness and behavior, back related health status, employment, and working conditions—could provide guidance on how individual WHP on back health interventions can be improved at the local level for providers and companies to better reach different target groups. However, this also applies to programs on a regional level. Furthermore, our findings are also useful for other countries, as back disorders are an overarching problem in the working population. However, not all factors with a potential effect on participation in WHP measures for back health, could be considered in this study. On one hand, these are missing information on the WHP offer itself like content or quality of the program. On the other hand, these are context factors at the workplace, for example the importance of work-related physical overload for the development of back disorders. High physical demands at the workplace, such as heavy lifting and carrying, can aggravate the symptoms of back pain, which in turn can increase the need to participate in back health measures. Such associations, as well as the influence of these and other relevant factors on the results of this work, thus remain unclear, but might interact with our analyzed factors. To better understand the use of WHP on back health, further research is necessary, particularly factors that promote and inhibit back health and the interactions between these factors on the individual and contextual level.

## Conclusion

Various studies have examined the growing spread of WHP offers for the promotion of back health among employees in Germany. However, the actual use and perception of these offers from the perspective of employees represents a relatively neglected field of research. Our study is one of the first in this research field to report the use of WHP from the users' perceptions. Furthermore, the special added value of our study is to present the first analyses of socio-demographic and -economic, work-, physical activity-, as well as health -related factors on the use of WHP on back health being representative for the population in Germany. Thus, it provides various aspects where to improve WHP on back health.

Our data indicated that different factors were relevant for women and men in taking advantage of company offers to promote back health. In addition to the finding that female employees were more likely to use offers than their male colleagues, women's participation was associated with the level of employment and health awareness. In contrast, for men, age, socioeconomic status, physical activity in leisure time and subjective back complaints were relevant factors in whether they made use of the services.

More frequent use by women working full-time highlights the need to address the needs of people with family and professional responsibilities. In addition, in view of the results among female employees, possible barriers due to reduced health awareness should be examined, and, if necessary, removed. Because the lowest utilization rate among male employees was in the 18–29 years age group, gender- and age-sensitive offers to promote back health in companies should also play a greater role in the future. For example, younger men may be more likely to focus on performance and competition than on other motivations and could be given greater consideration in the design of services (48). A frequently cited point of criticism regarding the prevention dilemma is that behavioral preventive measures to promote physical or sporting activity are often utilized by groups of the population that already have a practice of exercise-related behavior (14). The results of the current study highlight the importance of creating offers that also reach less physically or athletically active men. This could be achieved, for example, by applying particularly low-threshold concepts that consider people with different levels of experience with regard to physical or sporting activity, or individual incentive systems. In addition, it will be important for companies to strengthen positive attitudes toward health-promoting and preventive behavior (e.g., through healthy leadership behavior among employees). At the same time, this could also counteract the trend for many individuals, particularly male employees, to only take advantage of offers to promote back health if they already have back pain or a chronic back disease.

Further determinants of the use of WHP in general and back health offers should be studied. These include work-related psychosocial risk factors, social support in the company (including acceptance and support by managers), biomechanical workloads, occupational status, lack of time, service design, company size, expectations of self-effectiveness, and skills for self-motivation. The dissemination of quality standards in the design of WHP should then be promoted, as should the dissemination and use of company services to promote back health.

## Data Availability Statement

The datasets presented in this study can be found in online repositories. The names of the repository/repositories and accession number(s) can be found at: The GEDA 2014/2015-EHIS datasets on which the results are based are archived in the Research Agenda Center Health Monitoring at the Robert Koch Institute and are available to interested researchers on request. The dataset can be accessed on site in the Secure Data Center of the RKI's Health Monitoring Research Data Center. Inquiries can be made at the following e-mail address: fdz@rki.de.

## Author Contributions

SH wrote the manuscript and conducted the statistical analysis. AS, RG, and SJ assisted in the critical revision. SH, AS, and SJ designed the present study, developed the analysis plan, and interpreted the results. All authors read and approved the final manuscript.

## Conflict of Interest

The authors declare that the research was conducted in the absence of any commercial or financial relationships that could be construed as a potential conflict of interest.
